# Transcrestal Sinus Elevation with Implant Placement Using Autogenous Bone Supporting Multilayer Crosslinked Collagen Xenograft Scaffolding: A Case Series

**DOI:** 10.3390/dj14010064

**Published:** 2026-01-19

**Authors:** David Barack, Chander S. Gupta, Luigi Canullo, Marco Toia

**Affiliations:** 1 Private Practice, Skokie, IL 60076, USA; dangerdds@gmail.com (D.B.); cgupta17@gmail.com (C.S.G.); 2 Department of Prosthodontics, Unicamillus University, 00042 Rome, Italy; luigi.canullo@unicamillus.org; 3 Department of Oral and Maxillofacial Surgery and Oral Medicine, Faculty of Odontology, Malmö University, 21109 Malmö, Sweden

**Keywords:** transcrestal sinus lift, cross-linked collagen scaffold, autogenous bone, vertical bone gain, Schneiderian membrane, implant placement, atrophic maxilla

## Abstract

**Background/Objectives**: Limited residual bone height in the atrophic posterior maxilla complicates implant placement. Transcrestal sinus elevation can be used to correct bone shrinkage after sinus pneumatization or crestal bone loss. This study evaluated a minimally invasive, one-stage transcrestal sinus lift using a double-layer crosslinked collagen scaffold (MCCS) with autogenous bone from the implant osteotomy site in patients with RBH ≤ 6 mm. **Methods**: In this prospective series, 11 patients (48–64 years, mean RBH 4.75 mm, SD 0.95 mm) underwent one-stage transcrestal sinus floor elevation with simultaneous implants. After osteotomy, autogenous bone chips collected during drilling were compacted into the site, and two layers of MCCS were placed under the elevated Schneiderian membrane. Buccal and palatal bone heights were measured on CBCT before and after surgery to assess vertical bone gain (ΔRBH). **Results**: All implants achieved stable osseointegration. Mean ΔRBH was approximately 3.1 ± 0.9 mm (combined buccal–palatal). No postoperative complications occurred. Two small Schneiderian membrane perforations were sealed intraoperatively by MCCS placement, with uneventful healing. Follow-up imaging showed maintenance of the augmented bone around the implants. **Conclusions**: This double-layer MCCS plus autogenous bone approach is a safe, effective, and minimally invasive transcrestal sinus lift for atrophic maxillae. It yielded crestal bone gains even with minimal initial RBH, leveraging the palatal sinus wall’s osteogenic potential and the implant’s tent-pole effect. The MCCS scaffold maintained space for bone formation and enabled immediate sealing of any membrane perforations. This one-stage protocol is viable for implant placement in low-RBH sites.

## 1. Introduction

Achieving healthy and durable results when treating posterior maxilla edentulous sites with implants can be challenging due to limited residual bone height (RBH), poor bone quality, morbidity, operative risks, and cost [1,2,3,4]. Using bone substitutes in transcrestal or lateral sinus elevation approaches to fill the elevated space can increase vertical bone height [5,6,7,8]. Transcrestal sinus elevation can be used to correct bone shrinkage after sinus pneumatization or crestal bone loss [9,10]. In particular, transcrestal, rather than lateral, sinus elevation is suggested as an approach if RBH is 6–9 mm [7,11].

Different types of materials can be used to maintain the space created after lifting the Schneiderian membrane off the bone surface in sinus augmentation [4,12,13,14]. Among these, autogenous bone can be considered the gold standard grafting material due to its osteoconductive qualities [15]. However, use of autogenous bone is limited by graft resorption and donor site morbidity [16,17,18]. As a result, a variety of synthetic or animal-derived alternatives to autogenous bone, and techniques combining bone and alternatives to optimize osteoconductive and osteoinductive properties, have been developed. The combination of osteoconductive scaffold material and osteoinductive autogenous bone in transcrestal sinus elevation may have benefits compared with either alone, in terms of speed of healing of the posterior maxillary bone, optimizing natural bone, minimizing or eliminating residual graft material, and thereby reducing the risk of peri-implantitis [19,20]. Additionally, the placement of a flat, biodegradable barrier and biocompatible crosslinked collagen (MCCS) [21] beneath the sinus membrane can allow the sealing of potential perforations that may occur during the creation of an osteotomy. According to the manufacturers, as reported in previous studies, the MCCS is based on the proprietary material, which provides a volumizing, ossifying barrier membrane to allow the creation of greater volume of bone formation [22].

This case series introduces a transcrestal sinus elevation and implant placement protocol in which a double layer of MCCS is used in combination with autogenous bone collected during preparation of the implant osteotomy and describes the performance of the osteoconductive MCCS and osteoinductive autogenous bone in patients with RBH ≤ 6 mm.

## 2. Materials and Methods

Eleven implant placement procedures were performed in eleven patients (age 48–64 years) in maxillary premolar or molar sites in a private practice setting between January 2019 and November 2022. The study was conducted in accordance with the Declaration of Helsinki and approved by the Institutional Review Board: WCG, Association for the Accreditation of Human Research Protection Programs, Inc. (IRB Tracking Number: 20235092; Study Number: 1364166; Work Order Number: 1-1712930-1. Approval date: 13 November 2023). After having signed the informed consent, all of the patients presenting with missing either one or more teeth, requiring vertical sinus lift, and presenting with a residual bone height (RBH) of 4.73 ± 0.95 mm (sloped sinus floor) were treated. All patients included in this series were non-smokers, not diabetic, and free of any known autoimmune concerns (ASA 1) [23].

CBCT imaging (ProMax^®^ 3D Classic, Planmeca Oy, Helsinki, Finland) was taken for all patients prior to treatment. Measurements of the RBH were taken from cross-sectional images using a dedicated software (Romexis^®^, Planmeca Oy, Helsinki, Finland).

CBCT scans were acquired with a voxel size of 0.2 mm and a field of view covering the posterior maxilla. Linear measurements were made using the dedicated software, which allow marking points with 0.1 mm precision.

Linear measurements of the RBH were made from the coronal portion of the RBH to the edge of the sinus floor. Vertical measurements of the RBH were completed at the most mesial and distal points beneath the position of the implant. Buccal–palatal sinus width (SW) was measured to confirm sites remained within 12 mm dimensions [24]. Periapical measurements were also taken preoperatively, during the preparation of the osteotomy, and post implant placement. Follow-up images were recorded at various times dependent upon the availability of the patient following restoration of the implants (Table 1). Pre- and postoperative CBCT measurements were made on corresponding cross-sectional slices: the pre-op slice was chosen at the planned implant entry point, and the post-op slice was chosen along the implant axis. This ensured that buccal and palatal bone heights were measured at the same anatomical level All radiographic measurements were conducted by a single calibrated evaluator to ensure consistency.

After anesthesia, a flap was raised and osteotomies were performed with appropriate implant drills; drilling was to the sinus floor, often with partial exposure of the sinus membrane, and there was no intentional release of the sinus membrane with manual instruments. All sinuses were elevated with an MCCS (OSSIX^®^ Volumax, Dentsply Sirona, Charlotte, NC, USA), a biodegradable and biocompatible crosslinked collagen scaffold, intended for use during the process of guided bone regeneration (GBR) and guided tissue regeneration (GTR) as a biodegradable barrier [21], based on the proprietary material (GLYMATRIX^®^ collagen cross-linking technology, Dentsply Sirona, Charlotte, NC, USA) [22].

**Figure 1:**
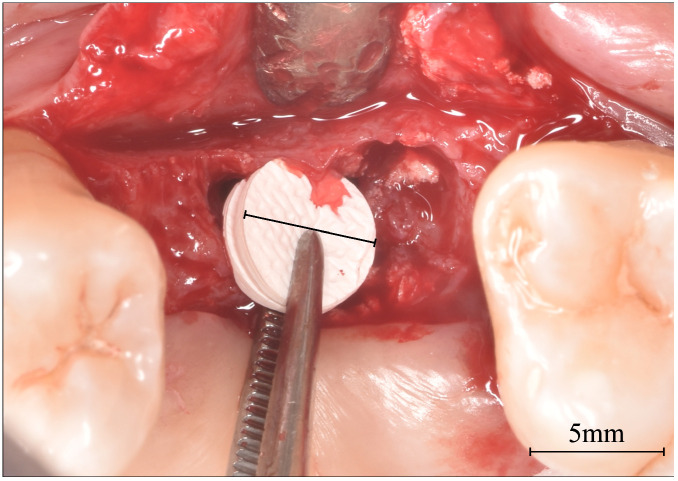
Crosslinked collagen scaffolds (MCCSs) were prepared using a 5 mm diameter biopsy punch for adaptation and placement into the osteotomy site.

**Figure 2:**
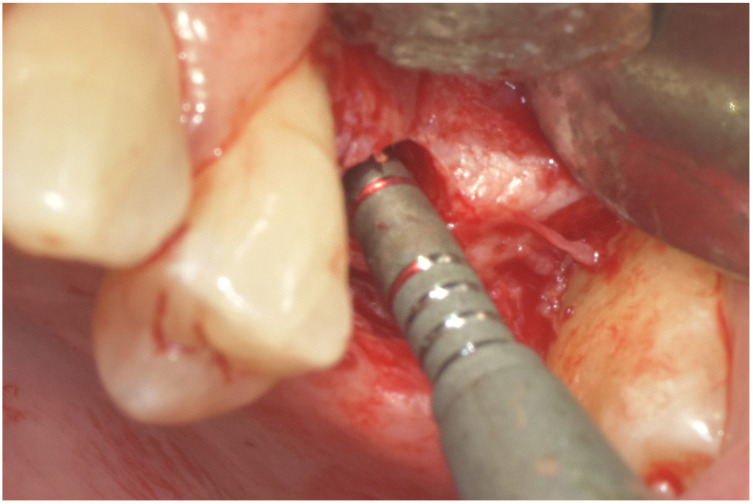
The crosslinked collagen scaffold (MCCS) was introduced into the osteotomy. A Summers osteotome was then used to carefully press and position the scaffold within the prepared site.

MCCSs were prepared using a 5 mm diameter biopsy punch (Figure 1); two layers of MCCS were punched, stacked together, and placed into the osteotomy site; a 4 mm or 5 mm Summer’s osteotome [9] was then used to position the MCCS immediately beneath the sinus floor. The MCCS was moistened by the patient blood from the surrounding osteotomy. Autogenous bone, collected with a bone harvesting suction filter device (Bone trap collector, DentsplySirona, Charlotte, NC, USA), was introduced to the osteotomy, placed with a curette at the orifice, and compressed to the top of the osteotomy with the Summer’s osteotome (Figure 2). The implant was then delivered to the site, which in turn pressed the autogenous bone and the MCCS superiorly until the implant was fully seated; a healing abutment or cover screw (two-staged protocol) was then threaded into place, and the site closed with sutures.

Outcomes were evaluated by change in RBH, measured radiographically using cone beam computed tomography (CBCT), at 4 months and at the follow-up visit. Measurements were made both along the buccal and palatal aspects of the implants. This was performed to allow for the variation in the sinus floor contours and sloped sinus floors. These measurements were compared to similar measurements off of the CBCT cross-sectional images. Landmarks were used to create fixed references for each case. Measurements were made to the tenth of a millimeter using a dedicated software (Romexis^®^, Planmeca Oy, Helsinki, Finland). Measurements of the length of implant penetrating beyond the original sinus floor were made using immediate postoperative periapical radiographs and were measured to the nearest half-millimeter using a dedicated software (Sidexis, Dentsply Sirona, Charlotte, NC, USA).

Descriptive statistics were computed for all continuous variables, including means, medians, standard deviations, minimum, and maximum values. These parameters were reported separately for buccal, palatal, and combined (mean buccal–palatal) measurements. Normality of distribution was assessed where appropriate. Differences between buccal and palatal measurements were analyzed using the Wilcoxon signed-rank test for paired data, given the non-parametric distribution of some variables. Statistical significance was defined as *p* < 0.05.

All analyses were performed using IBM SPSS Statistics for Windows, Version 29.0 (IBM Corp., Armonk, NY, USA).

## 3. Results

The demographic and implant characteristic are reported in Table 1.

**Figure 3:**
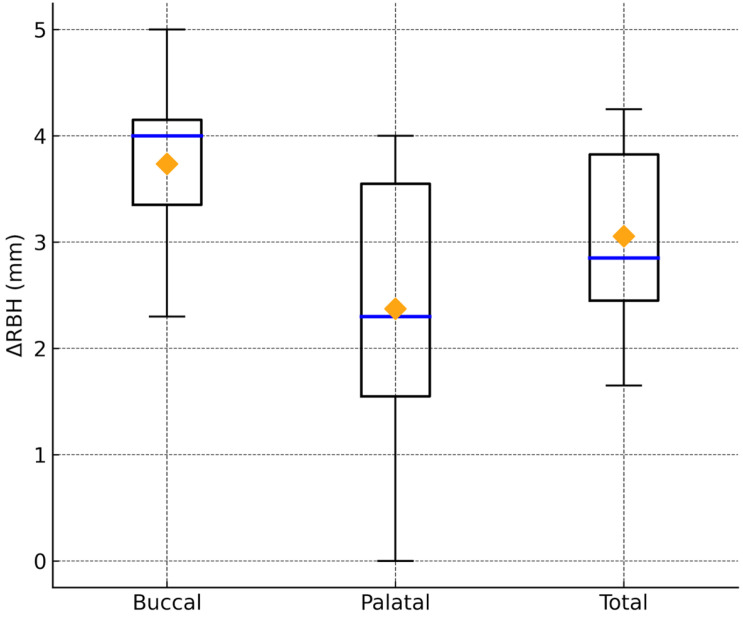
Boxplot illustrating vertical bone gain (ΔRBH = RBH_2_ − RBH) following lateral sinus augmentation with simultaneous implant placement. Measurements are reported separately at the buccal, palatal, and total (mean buccal–palatal) regions. The central horizontal line represents the median value, and the diamond marker indicates the mean. Whiskers denote the observed range. Buccal sites demonstrated greater vertical bone gain compared with palatal sites, while the combined value reflects the average augmentation achieved along the implant axis.

When ΔRBH was calculated arithmetically as RBH_2_ − RBH, buccal sites demonstrated significantly greater vertical bone gain compared with palatal sites. (Figure 3) (Table 2). The mean ΔRBH at the buccal aspect was 3.8 ± 0.8 mm, versus 2.4 ± 1.2 mm at the palatal aspect, resulting in an average difference of 1.4 mm (*p* = 0.0098, Wilcoxon signed-rank test). The combined buccal–palatal value showed a mean ΔRBH of 3.1 ± 0.9 mm.

**Figure 4:**
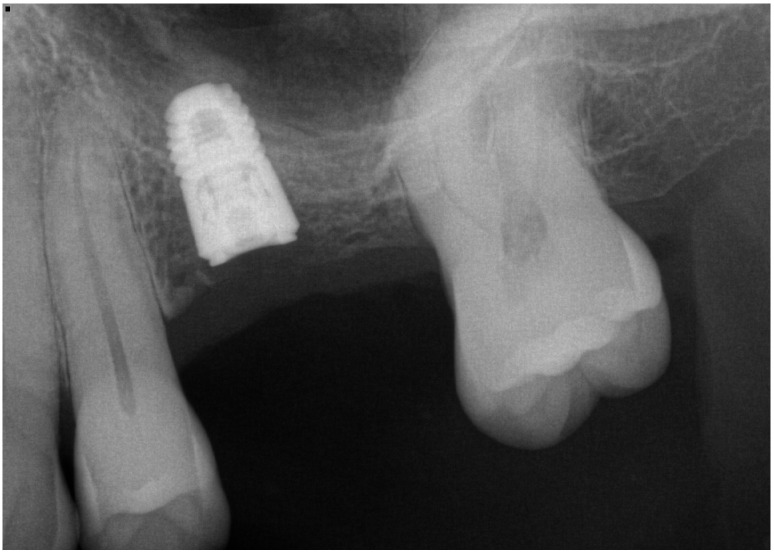
Periapical radiograph of Case #1 taken at the time of implant placement, demonstrating the elevation of the maxillary sinus.

**Figure 5:**
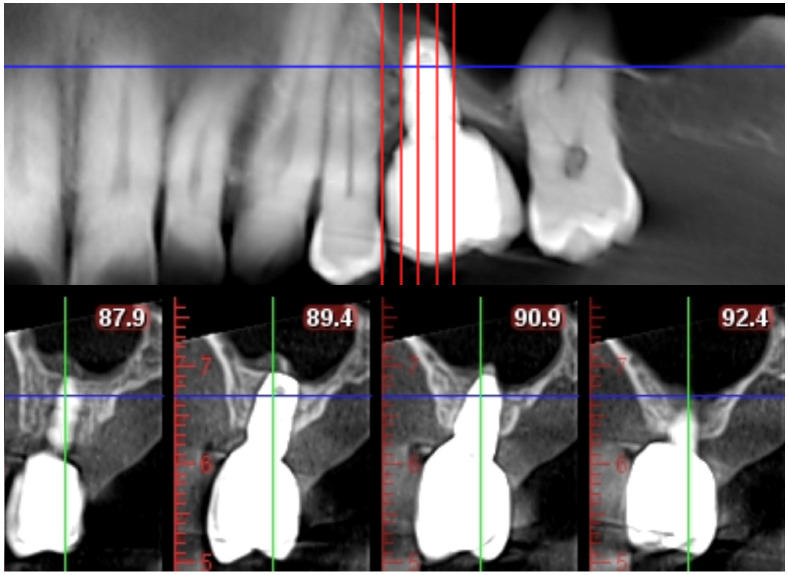
CBCT image of Case #1 at the 45-month follow-up, demonstrating stable implant integration.

Follow-up radiography showed consistent gain in bone height to the level of the implant apex, and CBCT (Figure 4 and Figure 5) revealed bone to the top of all the implants.

**Figure 6:**
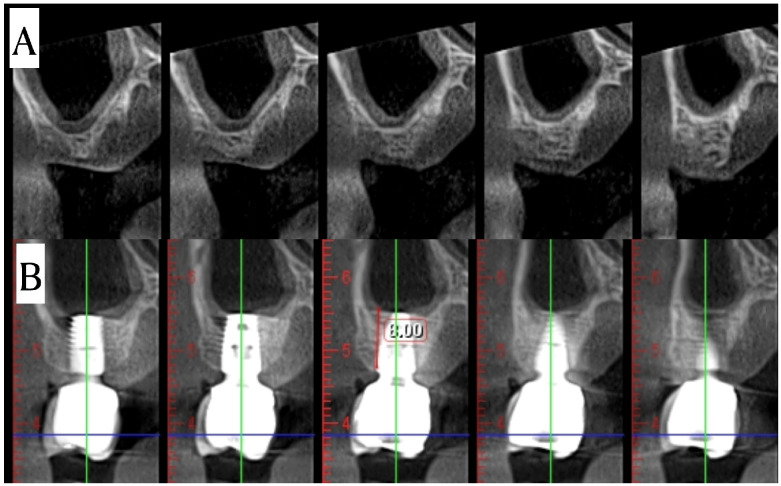
CBCT image of Case #5 at 10 months preoperatively (**A**) and postoperatively (**B**).

Results were consistent even with cases presenting with an initial crestal bone height with a mean of 3.35 mm (Figure 6).

**Figure 7:**
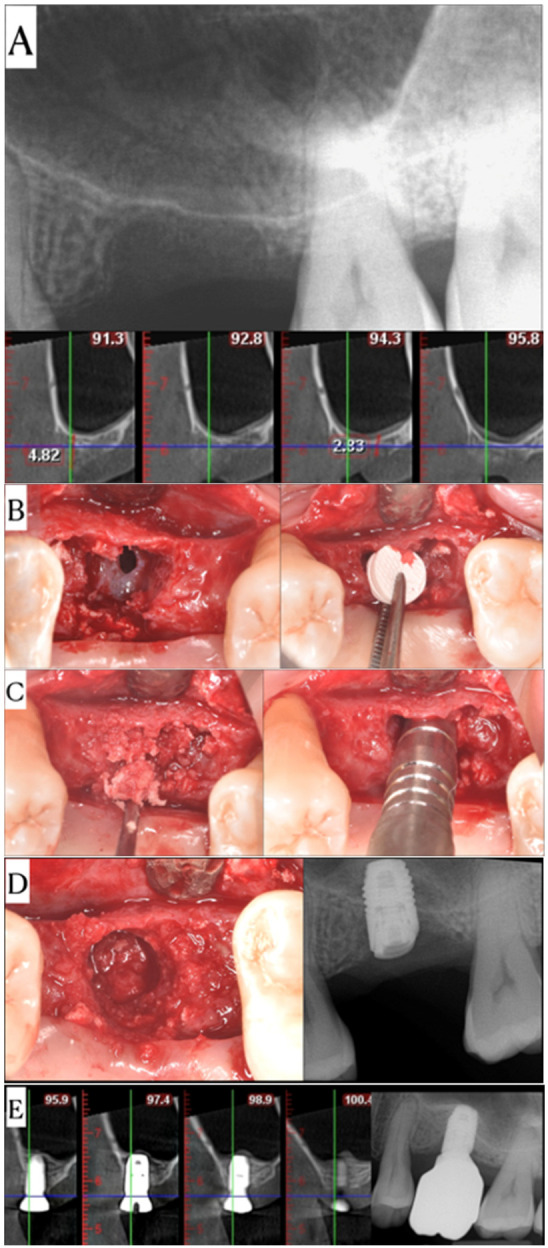
Sequential documentation of the surgical and healing phases of Case #11. (**A**) Preoperative CBCT coronal cross-section illustrates minimal residual maxillary sinus height, corroborated by the corresponding periapical radiograph. (**B**) Intraoperative visualization confirms Schneiderian membrane perforation, followed by placement of a crosslinked collagen scaffold (MCCS) to seal and protect the perforation. (**C**) Autogenous bone chips were inserted, and a Summers osteotome was used to gently compact the graft material and advance the membrane within the sinus cavity. (**D**) An occlusal clinical view demonstrates the autogenous bone in place prior to implant placement, with the postoperative periapical radiograph showing successful crestal sinus elevation. (**E**) A follow-up CBCT at 4.5 months reveals graft maturation around the implant, and the final periapical radiograph at 23 months demonstrates stable bone levels and successful implant integration.

Two cases were complicated by minor perforations of the sinus membrane and were sealed with the placement of the MCCS material against the membrane and directly over the perforation (Figure 7). No other adverse events were reported by the patients or observed clinically during the entire treatment period of these patients.

## 4. Discussion

The relative value of transcrestal sinus floor elevation techniques versus lateral-wall sinus floor elevation procedures and short implants continues to gain attention. While Thoma demonstrated the stability of short dental implants in maxillary posterior sites with crestal bone heights of 5–7 mm, other cases presenting to our care with less available RBH require sinus augmentation [25].

The present case series indicates that a transcrestal sinus elevation using a double-layer crosslinked collagen scaffold (MCCS) combined with autogenous bone chips can achieve observed vertical bone gain and implant success even in severely atrophic maxillae. We note that we do not claim any numerical superiority of the bilayer approach over a single-layer membrane; rather, our protocol was designed to maximize the predictability of bone regeneration in these challenging cases by combining the benefits of both components [26]. All 11 implants showed radiographic bone extending to their apices at follow-up, with a mean vertical bone gain of 3.1 mm. These outcomes are in line with the well-documented efficacy of transcrestal sinus floor elevation in augmenting limited posterior maxillary bone height, which several reviews report as comparable to lateral window sinus grafts in terms of bone gain and implant survival. Notably, transcrestal techniques offer clear advantages in minimally invasive management, as evidenced by significantly reduced patient morbidity, operative time, and postoperative discomfort relative to the lateral approach [27,28,29,30]. In our series, patients experienced uneventful recoveries with minimal pain or swelling, reflecting these advantages. This corresponds with Canullo et al.’s findings that crestal sinus augmentation is less painful and invasive (often requiring fewer analgesics) than lateral sinus lifts. In that 24-patient study, only one small membrane perforation occurred, with no graft migration or adverse effect, underscoring the safety of the transcrestal approach when properly executed [30].

Autogenous bone grafting played a central role in our protocol. Autogenous bone has long been considered the gold standard graft material due to its osteogenic and osteoinductive properties, providing living cells and growth factors that stimulate new bone formation [15].

However, conventional autografting often requires a secondary donor site, increasing patient morbidity and graft resorption risk [16,17,18]. In the present technique, we harnessed autogenous bone debris collected during osteotomy preparation—a strategy that adds osteoinductive potential without a separate harvest surgery. The volume of autograft from a single osteotomy is limited; therefore, placing the MCCS collagen scaffold above the bone chips served to amplify the graft volume and stabilize the clot under the sinus membrane. This combination leverages the rapid healing and osteogenic cell content of autograft together with the space-maintaining, osteoconductive scaffold, aiming for synergy in bone regeneration. Autologous bone, rich in osteoprogenitor cells and growth factors, provides an immediate osteogenic stimulus, while the MCCS collagen scaffold acts as a temporary matrix that stabilizes the blood clot, prevents soft-tissue invasion, and maintains space under the lifted membrane. The crosslinking of the collagen prolongs its resorption (on the order of 4–6 months), during which the scaffold can partially mineralize and integrate with new bone. Thus, the autograft jump-starts new trabecular bone formation, and the MCCS sustains the three-dimensional framework, together enabling guided bone regeneration in the sinus without additional graft materials [22]. Indeed, the concept of combining particulate autogenous bone with a collagen sponge is supported by prior research. Canullo et al. demonstrated that a sugar-crosslinked collagen sponge loaded with hydroxyapatite can promote true bone formation with minimal residual graft, yielding stable augmented volumes and high implant stability after 1 year [30]. They found that augmented bone height remained stable (no progressive resorption) up to 12 months, and importantly, patient-reported outcomes were very favorable (86% had no interference with chewing). These findings align with our clinical observations and suggest that a crosslinked collagen scaffold can be a reliable carrier for autogenous grafts, providing initial volume and gradually being replaced by new bone.

One remarkable property of the crosslinked collagen membrane used is its ability to ossify and integrate into newly formed bone. Zubery et al. histologically showed that a ribose-crosslinked collagen membrane in contact with native bone can become mineralized over a 5–6-month period [22]. In their human case series, new bone consistently formed along the membrane’s undersurface, and the membrane itself often demonstrated areas of calcification, essentially acting as an “ossifying barrier”. This suggests that the MCCS in our cases not only functioned as a passive scaffold but also actively participated in the regenerative process. The crosslinking delays membrane resorption for ~5–6 months, maintaining a barrier to soft tissue ingress while bone fills the subantral space. By the time the membrane fully resorbs, it may be partially replaced by bone. Such behavior was evident radiographically in our patients: the elevated sinus floor remained stable or even densified over time, with no voids or rapid collapse of the augmented space.

Another benefit of inserting a collagen scaffold beneath the Schneiderian membrane is the sealing of small membrane perforations should they occur. We intentionally placed one MCCS layer in direct contact with the lifted sinus mucosa, which in 2 of our 11 cases immediately served to cover a minor (<5 mm) perforation in the membrane. The second layer and bone graft were then added below this patch. In both cases, the perforation was effectively isolated from the sinus cavity, preventing graft leakage or infection, and normal bone regeneration ensued. This outcome is strongly supported by the literature. Studies have shown that sinus membrane perforations, if properly managed, do not preclude successful augmentation. Soares et al. (2024) conducted a meta-analysis of nearly 2000 perforated sinuses (ranging 2–15 mm) and found that when repaired, most commonly with a resorbable collagen membrane, bone formation and implant survival were equivalent to those in intact sinus cases. The overall implant loss in sites with repaired membranes was only ~4%, with no significant difference from intact sites and no correlation between perforation size and implant outcome [31]. Testori et al. likewise reported that even large membrane perforations could be successfully fixed using collagen membranes and sutures or pins, with implants placed as planned and survival rates comparable to non-perforated sinuses in the short term [32]. They emphasize key principles for perforation repair, such as ensuring the patch extends well beyond the perforation and is sufficiently stiff when wet to prevent sagging into the defect. In our transcrestal approach, the MCCS acted as an ideal repair patch: it is a multilayered, crosslinked collagen matrix that retains structural integrity when hydrated, allowing it to spackle a small sinus tear and support the membrane during elevation. This “internal bandage” effect prevented loss of graft particles into the sinus and likely averted sinusitis or other complications. It is worth noting that membrane perforation was the only intraoperative complication observed in most transcrestal lift studies, but its incidence is low and typically without lasting consequence when managed properly. Our experience corroborates this—minor perforations were infrequent (18% of cases) and effectively mitigated with the collagen scaffold, with no impact on final outcomes [32].

New bone formation in the sinus after transcrestal elevation appears to be influenced by both the technique and local anatomy. A consistent finding in our series is that the vertical bone gain is largely a function of the implant protrusion length beyond the original bone crest. Mainetti et al. reported a strong positive correlation (r ≈ 0.55) between the initial implant protrusion into the sinus and the amount of new bone formed around the implant apex. Over a mean 6.6-year follow-up of transcrestal augmentations, they found that each additional millimeter of implant extension yielded a proportional increase in bone gain, although a small portion of the implant apex (mean ~1.1 mm) often remained uncovered radiographically [33]. Similarly, Canullo et al. (2023) noted that the protruding implant length was the only significant predictor of graft height change in their 1-year study: neither residual bone height nor sinus width influenced the outcome, but longer implant extension correlated with greater maintained graft height at 6 and 12 months [30]. These findings support the clinical intuition that an implant acts as a tent pole under the sinus membrane, supporting a larger space for bone infill.

The osteogenic potential of the maxillary sinus walls plays a critical role in bone formation following sinus augmentation, with the palatal wall consistently described as a more favorable biological source of osteoprogenitor cells and vascular supply [34]. In accordance with these biological principles, our clinical series demonstrated a statistically significant difference in the vertical bone gain of 3.1 mm. Specifically, the buccal surface exhibited an average of vertical bone gain that was approximately 1.36 mm statistically greater than the palatal surface. This observation supports the concept that the palatal wall acts as a primary osteogenic and angiogenic contributor during healing, providing a more robust environment for clot maturation and scaffold transformation.

These combined mechanisms likely contributed to the differential bone formation observed in our cohort. The implant’s presence may also promote mechanotransductive stimulus and angiogenesis along its active surface [35], encouraging bone to creep up toward the apex.

However, there is a practical limit to this strategy. If an implant is over-extended without sufficient primary stability, it risks micromovement and failure. In one of Tsai et al.’s transcrestal cases with RBH ≤ 3 mm, an implant placed with excessive protrusion (and inadequate initial stability) failed early, whereas the vast majority integrated successfully. In their study the authors reported on one-stage vs. two-stage sinus lifts in extremely atrophic maxillae. Despite a mean residual bone of only 3 mm, transcrestal augmentation achieved an overall 98.9% implant survival, statistically equivalent bone gains to lateral window grafting, and no prosthetic failures over 4–6 years. Notably, Tsai et al. found that a staged transcrestal approach (sinus lift with graft, followed by delayed implant placement) yielded outcomes on par with lateral sinus lift but with lower invasiveness, whereas one-stage transcrestal surgery offered the quickest rehabilitation at the cost of slightly higher risk. They concluded that transcrestal sinus elevation—even in extremely low bone—can be a viable alternative to lateral surgery, provided careful case selection and surgical expertise [28]. In the present clinical report, all cases had at least 2–3 mm of native bone which allowed simultaneous implant placement with adequate primary stability.

The osteogenic potential of the Schneiderian membrane and surrounding bony walls in a closed sinus environment should not be underestimated. The transcrestal technique essentially creates a secluded chamber above the native bone, which quickly fills with blood and inflammatory cells, forming a coagulum rich in growth factors. The elevated sinus membrane (with intact periosteum) and the adjacent cortical walls constitute a source of osteoprogenitor cells that can migrate into the clot.

It has been shown that even without any graft material, simply lifting the membrane can result in appreciable bone formation. For example, Lafzi et al. observed an average 4 mm of bone gain after osteotome sinus elevation without grafting, with maintained implant success at 10 years [36]. Similarly, a graftless hydraulic sinus lift using only saline or platelet-rich fibrin can produce some new bone (albeit less than with a bone graft) while completely avoiding foreign material. Farina et al.’s systematic review confirms that the use of a graft vs. graftless approach has minimal impact on complication rates or implant survival in transcrestal procedures [29]. Where grafts do make a difference is in the volume of bone augmented: several studies report that adding a particulate graft (xenograft, allograft, or autograft) under the membrane leads to a greater vertical bone fill and a higher likelihood that the implant apex will be completely encased in new bone, especially over the long term [4,12,13,14]. In our view, autogenous bone is particularly advantageous in this context because it not only serves as a scaffold but also actively induces bone formation, and it is eventually replaced by vital bone rather than remaining as residual particles. The contiguous native bone walls (floor and lateral walls of the sinus) in combination with the autograft contribute osteogenic cells that drive what is essentially an in situ guided bone regeneration. The MCCS membrane further enhances this by containing the graft and clot within the desired space and preventing fibrous tissue ingrowth. This principle of creating a confined regenerative space is a cornerstone of transcrestal sinus lift philosophy and is a reason why minimal invasiveness can still yield optimal outcomes.

The thick, multilayer crosslinked collagen scaffold provides a space-maintaining, biodegradable matrix (with a compact “barrier” layer and porous inner layers) that resists collapse and promotes vascular ingrowth. Autogenous bone chips, in turn, supply viable osteogenic cells (osteoblasts and progenitors) and growth factors (e.g., BMPs and TGF-β) that accelerate new bone formation. Together, they create a tent-pole effect with the implant and palatal cortical wall, forming a confined regenerative chamber for bone formation [37].

Finally, it is important to contextualize our technique among the vast transalveolar sinus lift modifications described in the literature. Since Tatum’s and Summers’ seminal work [9,10], clinicians have continuously refined the transcrestal approach to improve safety and predictability [36,38,39]. Techniques now range from purely manual osteotome-mediated lifts to drill-assisted approaches, motorized reamers, piezoelectric crestal windows, hydraulic balloon-assisted lifts, and more. Notably, all such transcrestal techniques are less invasive and faster than the lateral window, and whenever feasible, performing a one-stage sinus lift with simultaneous implant placement is preferred to reduce treatment time and patient burden. The present protocol fits well within this modern paradigm of minimally invasive implant reconstruction. It requires only a standard implant osteotomy (plus use of an osteotome for final sinus membrane elevation) and makes use of novel biomaterials (a crosslinked collagen sponge) to enhance outcomes without adding morbidity. Our results add to the growing evidence that with careful technique, transcrestal sinus augmentation can reliably rehabilitate atrophic posterior maxillae with reduced risk and high success rates. Despite its promising outcomes, this study has several limitations. First, the sample size was small (only 11 cases), which limits the generalizability of the findings. Second, all surgeries were performed at a single clinical center, so the results may not readily translate to other clinical settings or operators. Third, the patient population was homogeneous, consisting exclusively of ASA I individuals (healthy patients with no systemic conditions), which means these outcomes might not be directly applicable to patients with more complex medical histories. A fourth limitation concerns the heterogeneous follow-up durations of the study. This protocol is intended only for carefully selected cases with favorable anatomical conditions and healthy patients, given the intrinsic difficulty of augmenting sites with extremely low residual bone height.

## 5. Conclusions

This case series highlights that employing two layers of a crosslinked collagen scaffold in conjunction with autogenous bone from the implant site is a clinically effective strategy for transcrestal sinus floor elevation in cases of minimal residual bone.

- A mean vertical bone gain of 3.1 ± 0.9 mm was achieved with the transcrestal sinus lift using a double-layer crosslinked collagen membrane.
- No postoperative complications were observed in any patient, indicating a favorable safety profile for this technique.
- The transcrestal sinus lift approach proved effective even in cases with minimal residual bone height, demonstrating its utility in challenging atrophic maxillary scenarios.
- The crosslinked collagen membrane played a crucial role in space maintenance and supported new bone regeneration, while also facilitating the closure of any sinus membrane perforations.
- These results are consistent with the current literature, which supports the efficacy and safety of the transcrestal sinus lift technique with collagen membrane augmentation.

## Figures and Tables

**Table 1:** Demographic and clinical characteristics, including case number, patient age, implant type, and residual bone composition at the implant site.

Case	Age (Years)	Site UNS ^a^	Protocol	Implant Type	SW ^b^ (mm)	RBH ^c^ (b-p mm)	LIP ^d^ (b-p mm)	RBH2 ^e^ (b-p mm)	F-U ^f^ (Months)	∆RBH ^g^ (b-p mm)
1	64	14	No	DS ^i^ Profile EV	10.2	3.8–5.8	2.0–3.8	6.2–8.0	57	2.4–2.2
2	61	4	No	DS ^i^ Astra Tech EV	11.6	3.8–5.0	2.0–4.0	6.1–8.0	48	2.3–3.0
3	61	15	No	DS ^i^ Profile EV	12.4	3.4–4.1	5.0–6.0	7.6–8.1	4	4.2–4.0
4	60	3	Yes	DS ^i^ Prime Taper EV	8.6	3.2–6.2	5.5–3.5	7.8–8.1	19	4.6–1.9
5 ^h^	51	3	Yes	DS ^i^ Prime Taper EV	8.0	4.0–7.0	2.0–2.5	8.0–8.2	10	4.0–1.2
6	64	3	Yes	DS ^i^ Prime Taper EV	10.4	4.2–8.0	4.0–4.5	8.0–8.8	10	3.8–0.8
7	65	3	Yes	Biohorizon	11	4.5–5.4	3.5–4.0	8.6–9.0	7	4.1–3.6
				Tapered Pro						
8	63	14	Yes	DS ^i^ Prime Taper EV	7.6	4.7–8.0	0.0–3.0	8.0–8.0	8.5	3.3–0.0
9	64	3	Yes	DS ^i^ Prime Taper EV	11.2	3.0–5.1	4.0–4.5	8.0–8.6	6	5.0–3.5
10	48	4	Yes	Biomet 3i Prevail	8.4	3.4–4.8	2.0–4.5	6.8–7.1	6	3.4–2.3
11 ^h^	52	14	Yes	DS ^i^ Profile EV	11.1	2.2–4.5	3.0–6.0	6.2–8.1	4.5	4.0–3.6

^a^ Universal Numbering System (UNS); ^b^ Sinus width (SW) measured from the buccal to the palatal wall. ^c^ Residual bone height (RBH), buccal–palatal (b-p mm) before the surgery. ^d^ Length of implant (LIP) penetrating into the sinus. ^e^ Residual bone height (RBH2), buccal–palatal (b-p mm) at the follow-up visit. ^f^ Follow-up (F-U) reported in months. ^g^ Vertical bone gain (∆RBH), buccal–palatal (b-p mm), between before and after the surgery. ^h^ The Schneiderian membrane was perforated during the osteotomy. ^i^ Dentsply Sirona (DS) (EV family implants, Dentsply Sirona, Charlotte, NC, USA).

**Table 2:** Summary of descriptive statistics for residual bone height (RBH, mm), postoperative bone height (RBH2, mm), and vertical bone gain (ΔRBH = RBH2 − RBH, mm), reported for buccal, palatal, and total (mean buccal–palatal) measurements. Values are presented as minimum, maximum, mean, median, and standard deviation.

Variable	Site	Min	Max	Mean	Median	Std
RBH	Buccal	2.2	4.7	3.7	3.8	0.7
RBH	Palatal	4.1	8	5.8	5.4	1.4
RBH	Total	3.4	6.4	4.7	4.7	1.0
RBH2	Buccal	6.1	8.6	7.4	7.8	0.9
RBH2	Palatal	7.1	9	8.3	8.1	0.5
RBH2	Total	7.0	8.8	7.8	8.0	0.6
ΔRBH	Buccal	2.3	5	3.8 **	3.8	0.8
ΔRBH	Palatal	0	4	2.4 **	2.3	1.2
ΔRBH	Total	1.2	4.5	3.1 **	3.0	0.9

** A paired Wilcoxon signed-rank test demonstrated a statistically significant difference between buccal and palatal ΔRBH values (*p* = 0.0098), indicating greater vertical bone gain at the buccal aspect when ΔRBH was calculated arithmetically as RBH_2_ − RBH.

## Data Availability

No data is available due to privacy.

## Abbreviations

The following abbreviations are used in this manuscript:

UNS	Universal Numbering System
SW	Sinus width
RBH	Residual bone height
∆RBH	Vertical bone gain
MCCS	Crosslinked collagen scaffold
